# The Neuroprotection of Low-Dose Morphine in Cellular and Animal Models of Parkinson’s Disease Through Ameliorating Endoplasmic Reticulum (ER) Stress and Activating Autophagy

**DOI:** 10.3389/fnmol.2018.00120

**Published:** 2018-04-20

**Authors:** Bing Wang, Cun-Jin Su, Teng-Teng Liu, Yan Zhou, Yu Feng, Ya Huang, Xu Liu, Zhi-Hong Wang, Li-Hua Chen, Wei-Feng Luo, Tong Liu

**Affiliations:** ^1^Jiangsu Key Laboratory of Neuropsychiatric Diseases and the Second Affiliated Hospital of Soochow University, Suzhou, China; ^2^Institute of Neuroscience, Soochow University, Suzhou, China; ^3^Jiangsu Key Laboratory of Preventive and Translational Medicine for Geriatric Diseases, Department of Nutrition and Food Hygiene, School of Public Health, Soochow University, Suzhou, China

**Keywords:** morphine, Parkinson’s disease, neuroprotection, autophagy, ER stress

## Abstract

Parkinson’s disease (PD) is a common neurodegenerative disease characterized the progressive loss of dopaminergic (DA) neurons in the substantia nigra pars compacta (SNc). Brain endogenous morphine biosynthesis was reported to be impaired in PD patients and exogenous morphine attenuated 6-hydroxydopamine (6-OHDA)-induced cell death *in vitro*. However, the mechanisms underlying neuroprotection of morphine in PD are still unclear. In the present study, we investigated the neuroprotective effects of low-dose morphine in cellular and animal models of PD and the possible underlying mechanisms. Herein, we found 6-OHDA and rotenone decreased the mRNA expression of key enzymes involved in endogenous morphine biosynthesis in SH-SY5Y cells. Incubation of morphine prevented 6-OHDA-induced apoptosis, restored mitochondrial membrane potential, and inhibited the accumulation of intracellular reactive oxygen species (ROS) in SH-SY5Y cells. Furthermore, morphine attenuated the 6-OHDA-induced endoplasmic reticulum (ER) stress possible by activating autophagy in SH-SY5Y cells. Finally, oral application of low-dose morphine significantly improved midbrain tyrosine hydroxylase (TH) expression, decreased apomorphine-evoked rotation and attenuated pain hypersensitivity in a 6-OHDA-induced PD rat model, without the risks associated with morphine addiction. Feeding of low-dose morphine prolonged the lifespan and improved the motor function in several transgenic *Drosophila* PD models in gender, genotype, and dose-dependent manners. Overall, our results suggest that neuroprotection of low-dose morphine may be mediated by attenuating ER stress and oxidative stress, activating autophagy, and ameliorating mitochondrial function.

## Introduction

Parkinson’s disease (PD) is the second most common neurodegenerative disease, which is characterized by the progressive loss of dopaminergic (DA) neurons in the substantia nigra compacta (SNc). A hallmark of PD is the presence of Lewy’s bodies in neurons, which is the cytoplasmic inclusion mainly comprised of aggregated α-synuclein (Kalia and Lang, 2015). Although the etiology of PD remains obscure, it is now appreciated that genetic and environmental factors contribute to the pathogenesis of PD. Mounting evidence indicated that damage of DA neurons may arise from oxidative stress (Jiang et al., 2016), autophagy-lysosomal dysfunction (Button et al., 2015; Wu et al., 2015; Menzies et al., 2017), endoplasmic reticulum (ER) stress (Celardo et al., 2016; Mercado et al., 2016), mitochondrial dysfunction (Abou-Sleiman et al., 2006), and disruption of calcium homeostasis (Parlato and Liss, 2018). To date, although levodopa is widely used as the most effective drug for symptomatic treatment of PD, there is no medication to slow down the progression of PD. Therefore, the development of neuroprotective therapies to slow the progression of PD is necessary for treatment of PD.

Endoplasmic reticulum stress is a pathological sate that caused by the aberrant accumulation of misfolded or unfolded proteins in its lumen (Senft and Ronai, 2015). Transient ER stress activates intracellular signal transduction pathways collectively termed the unfolded protein response (UPR), which is a physiological protective response aimed to restore ER homeostasis (Halliday and Mallucci, 2014). The UPR is mediated by the activation of three main stress sensors located at the ER membrane, including inositol requiring kinase1a (IRE1a), protein kinase RNA-like kinase (PERK), and activating transcription factor 6 (ATF6) (Hetz, 2013). The activation of these signaling pathways controls the expression of genes that involved in the secretory pathways and results in the reduction of misfolded proteins at the ER. However, under chronic ER stress, accumulation of misfolded proteins and persistent UPR activates the pro-apoptotic pathways and cell death. Signs of chronic ER stress and highly expressed genes involved in the UPR were consistently observed in the post-mortem brain from PD patients and in animal models of PD, suggesting chronic ER stress contributes to pathology of PD (Matus et al., 2011). Furthermore, it was shown that accumulation and aggregation of α-synuclein was critical for triggering deleterious chronic ER stress (Guardia-Laguarta et al., 2015). Thus, targeting ER stress may be a promising neuroprotective strategy for management of PD.

Endoplasmic reticulum stress and autophagy display complex interactions and is implicated in the pathology of many neurodegenerative diseases. Autophagy is a self-destructive process to remove misfolded or aggregated proteins and/or damaged organelles (e.g., mitochondria) via lysosomal degradation. Mounting evidence supports that autophagy-lysosomal dysfunction is involved in the pathogenesis of PD (Xilouri et al., 2016). Given aggregated α-synuclein is mainly degraded via autophagy (Xilouri et al., 2016), autophagy dysfunction causes aberrant accumulation of aggregated α-synuclein. Moreover, autophagy dysfunction is also induced by PD-related neurotoxins, such as 6-hydroxydopamine (6-OHDA), rotenone, or 1-methyl-4-phenyl-1, 2, 3, 6-tetrahydropyridine (MPTP) in cellular and animal models (Wu et al., 2015; Lu et al., 2016). Autophagy ameliorates ER stress by eliminating misfolded and/or aggregated proteins (Han et al., 2015). Recent studies have shown that appropriate up-regulation of autophagy appears to be neuroprotective due to clearing aggregated α-synuclein in cellular and animal PD models (Xilouri et al., 2016). Thus, the autophagy enhancer may be beneficial to slow the disease progression of PD.

Morphine is an alkaloid from the plant extracts of the opium poppy (Diamond and Desgagné-Penix, 2016). Clinically, morphine is widely used as a strong analgesic to treat chronic pain, such as postoperative pain, neuropathic pain and cancer pain (Bruel and Burton, 2016; Cooper et al., 2017). High dose or long-term application of morphine may also produce side effects, such as constipation, respiratory depression, and immunosuppression (Qiu et al., 2015). In the central nervous system (CNS), morphine has been shown to regulate cell death or survival of neurons and astrocytes (Zhang et al., 2016). To date, either destructive or protective effects of morphine on neurons in the CNS have been reported (Zhang et al., 2008). Exposure of morphine is detrimental for synaptic densities in hippocampal neurons (Cai et al., 2016). Recently, it was reported that morphine triggered Atg5- and Atg7-dependent autophagy in dopaminergic neurons and contributed to reward, behavioral sensitization, analgesic tolerance and physical dependence (Su et al., 2017). In contrast, there is also evidence that morphine shows neuroprotective effects in the CNS. For instance, morphine protected SH-SY5Y cells against 6-OHDA-induced cell damage possible via anti-oxidant and calcium blocking mechanisms (Elyasi et al., 2014). Morphine also prevented SK-N-SH cells injury induced by rotenone possible through the modulation of the ubiquitin-proteasome complex (Rambhia et al., 2005). Morphine protected neurons against neurotoxicity induced by Aβ oligomers, which is involved in the pathogenesis of Alzheimer’s disease (Wang Y. et al., 2015). Thus, these results indicated that morphine produced either detrimental or protective effects in the CNS and the effects of morphine may be dependent on the dosage and exposure time. Interestingly, compelling evidence demonstrated that endogenous morphine exists and is biosynthesized in the CNS (Guarna et al., 2005) and is able to regulate dopaminergic neurotransmission (Stefano et al., 2012). Moreover, dopamine is demonstrated to be the precursor for endogenous morphine biosynthesis (Stefano et al., 2008). Intriguingly, previous study indicated that endogenous morphine systems were reciprocally dysregulated in PD (Stefano et al., 2012). Together, these studies indicated low level of endogenous morphine may be protective for neurodegenerative disease, particular PD. However, the mechanisms underlying neuroprotection of low dose morphine in PD are still unclear.

The present study was performed to investigate the neuroprotection of low-dose morphine in cellular and animal PD models and possible underlying mechanisms. Herein, we demonstrated that morphine significantly protected SH-SY5Y cells against cell damage induced by 6-OHDA or rotenone. Furthermore, we found morphine attenuated ER stress and oxidative stress, activated autophagy, and improved mitochondria function in 6-OHDA-treated SH-SY5Y cells. Meanwhile, we found oral application of low-dose morphine improved midbrain tyrosine hydroxylase (TH) expression, motor deficits and pain in a 6-OHDA-induced PD rat model, without signs of physical dependence. Finally, feeding of low-dose morphine prolonged the lifespan and improved the motor function in several transgenic *Drosophila* PD models in gender, genotype, and dose-dependent manners. Together, our results suggested application of low-dose morphine may play a neuroprotective role in PD without risk of physical dependence.

## Materials and Methods

### Animals and Treatment

Adult male Sprague-Dawley (SD) rats (200–220 g) were obtained from the SLAC Laboratory Animal Co., Ltd. (Shanghai, China). Animals were kept in controlled room temperature (22 ± 2°C) and humidity (60–80%) under a 12 h/12 h light/dark cycle and housed with food and water available *ad libitum*. The rat model of PD was established by micro-injection of 6-OHDA (3 μl, 2 μg/μl) into the right medial forebrain bundle at the following rate (coordinates: A: -1.8 mm from bregma, L: -2.5 mm from midline and H: -7.5 mm). The sham rats were treated with saline at the same location. Morphine (0.01 mg/ml and 0.05 mg/ml) was added to the drinking water in rats. All experimental procedures and animal handing were performed in accordance with the guidelines of the International Association for the Study of Pain and the animal protocols were approved by Soochow University Animal Committee. All toxic reagents in this study were approved by the Medical Laboratory Center of Soochow University.

All *Drosophila* stocks were cultured in standard media and maintained at 25°C incubator on a 12 h/12 h light/dark cycle. *UAS-synuclein-WT*, *UAS-LRRK2-G2019S*, *UAS-synuclein-A53T*, *UAS-Parkin-Q311X*, *Elav-GAL4*, and *Ddc-GAL4* were obtained from the Bloomington Stock Center (Indiana University, United States). The *Elav-synuclein-WT*, *Elav-synuclein-A53T*, *Elav-Parkin-Q311X (Elav-Q311X)*, and *Ddc-LRRK2-G2019S (Ddc-GS2)* transgenic PD files were obtained by hybridizing the above *Drosophila* strains. The final concentration of rotenone added to the food was 100 μM. The transgenic PD files and the rotenone-induced PD flies were feed with morphine (0.01 mg/ml, 0.05 mg/ml, and 0.25 mg/ml). The standard biosecurity and institutional safety procedures been carried out for handling biohazards, biological select agents, toxins, restricted materials or reagents.

### Apomorphine-Induced Rotation

One to five weeks after 6-OHDA injection, apomorphine was injected subcutaneously (0.5 mg/kg). The number of rotations (turns/min) was recorded and counted for 30 min.

### Rotarod Test

We used the rotarod apparatus to assess the motor function. Rats were trained for 3–4 days before testing, with increased speed from 0 to 20 rpm in 2 min, until rat kept steadily performance. All rats were tested at 20 rpm for 5 min with an interval of 20 min for 3 trials and the average latency for falling was recorded and analyzed.

### Catalepsy Test

The forelimbs of the rat were placed on a 9 cm high bar and the latency of the forelimb movement was recorded by a stopwatch.

### Naloxone-Precipitated Withdrawal Response Test

The rats were divided into three groups: (1) Normal group of rats; (2) Morphine addiction rats was induced by repeated injection of morphine as previously described. Morphine was intraperitoneally given twice every day at am 9:00 and pm 6:00 for 5 days and was gradually increased as follows: 1st day, 20 mg/kg; 2nd day, 40 mg/kg; 3rd day, 60 mg/kg; 4th day, 80 mg/kg; 5th day, 100 mg/kg. 6th day, rats were injected with 100 mg/kg morphine before precipitating withdrawal symptoms. (3) Treatment of morphine (0.01 mg/ml and 0.05 mg/ml) in 6-OHDA-injected rats. Withdrawal symptoms of three groups were precipitated 2 h later by injection of naloxone (1 mg/kg, subcutaneous). The rats were habituated for 30 min to a beaker (3L) before injection of naloxone. Naloxone-precipitated withdrawal responses were observed for 30 min, including salivation, teeth chattering, body tremor and diarrhea. The global withdrawal score was calculated for each rat. A score was given for each withdrawal sign and global withdrawal score was calculated (maximum score is 6).

### Survival Curve of Flies

Twenty to twenty five newly enclosed male or female flies were collected in a plastic tube (15 cm in length and 1.5 cm in diameter) with food. The flies were moved into the tubes with fresh food every other day. The survival of flies was recorded every day. Based on survival curve of flies, we used the 50% survival time to compare the survival ratio between the different groups. We performed at least 3 independent experiments.

### Climbing Assay in Flies

To analyze the locomotor ability of flies, the negative geotaxis assay was used (Liu et al., 2008). Briefly, a cohort of 20–25 flies from each group was performed the climbing assay weekly, and the tested flies were moved to a vertical plastic tube (15 cm in length and 1.5 cm in diameter). After the flies were habituated for 30 min at room temperature, and then were gently rapped to the bottom of tube. The number of flies that could climb to the test line within 10 s was counted. We used the half-pass time to compare the climbing abilities between the different groups, which means time at which 50% of the flies were able to climb above the test line. We performed at least 3 independent experiments.

### SH-SY5Y Cell Culture

The human neuroblastoma SH-SY5Y cells were cultured in Dulbecco modified Eagle’s medium (DMEM) supplemented with 10% FBS, penicillin G (100 U/mL) and streptomycin (0.1 mg/mL). Cells were cultured at 37°C in a 5% CO_2_ atmosphere. The SH-SY5Y cells were seeded at the density of 3000 cells/well in 96-well microplate for the MTT assay, measurement of mitochondrial membrane potential, intracellular calcium levels, and reactive oxygen species (ROS) assays. The cells were incubated with 6-OHDA and different concentration of morphine for 24 h. Morphine was added 24 h before 6-OHDA. The ER stress inhibitor 4-phenylbutyrate (4-PBA) and autophagy inhibitor 3-Methyladenine (3-MA) were added 1 h before incubation of morphine.

### Cell Viability Analysis

Cellular viability was analyzed by MTT Cell Proliferation and Cytotoxicity Assay Kit. MTT was dissolved in MTT solvent at a final concentration of 0.5 mg/ml. According to experimental, cultured and gave specific drug stimulation. Added 10 μl of MTT solution to each well and incubated for another 4 h at 37°C, then the medium was carefully removed and 100 μl dimethylsulfoxide (DMSO) was added to each well. The absorbance values were determined at 570 nm with an automatic microplate reader.

### Annexin V-FITC Apoptosis Detection

The cell apoptosis was detected by the Annexin V-FITC/PI double staining. Briefly, cells were collected by trypsinization, and washed twice with cold 0.01 M phosphate buffer (pH 7.4), then re-suspended in binding buffer. The cells were mixed after adding 5 μl Annexin V-FITC, incubated for 15 min at room temperature in the dark. Then, 5 μL PI was added for 5 min. Finally, added 200 μl binding buffer, and analyzed by flow cytometry.

### Measurement of Intracellular ROS

The intracellular ROS were determined by a 2, 7-dichlorofluorescein diacetate (DCFH-DA) probe. Briefly, cultured SH-SY5Y cells were seeded in 24-well or 6-well plates at least 12 h before treatment. The treated cells were washed with 0.01 M phosphate buffer (pH 7.4) for 15 min. Cells were incubated in DCFH-DA (25 μM) for 30 min at 37°C, then washed three times with cold 0.01 M phosphate buffer (pH 7.4). The fluorescence was detected with a Zeiss fluorescence confocal microscope LSM700 (Oberkochen, Germany), and images were treated with ZEN software or Adobe Photoshop (San Jose, CA, United States).

### Measurement of Intracellular Ca^2+^ Levels

The treated cultured SH-SY5Y cells in 24-well plates were incubated with 10 μM Fluo-3/AM for 60 min at 37°C. Cells were then washed with Ca^2+^-free PBS for three times to remove the extracellular fluo-3/AM, and then 200 μl DMEM was added. A confocal laser scanning microscope (Leica, Germany) recorded the fluorescence images indicating the Ca^2+^ concentration.

### Measurement of Mitochondrial Membrane Potential

The mitochondrial membrane potential was determined with mitochondrial membrane potential assay kit with JC-1. JC-1 is an ideal fluorescent probe that is widely used to detect the mitochondrial membrane potential ΔΨm. At higher mitochondrial membrane potential, JC-1 aggregates in the matrix of mitochondria to form J-aggregates that produce red fluorescence; JC-1 can’t accumulate in the mitochondria at lower mitochondrial membrane potential. In this case, JC-1 is a monomer that produces green fluorescence. This makes it very easy to detect the changes in mitochondrial membrane potential by the change of fluorescence color. Collected 10–60 million cells, re-suspended in 0.5 ml cell culture medium. JC-1 staining working solution (0.5 ml) was added, and then incubated for 20 min at 37°C. After incubation, cells were centrifuged at 600 *g* for 3–4 min at 4°C to pellet the cells. Cells were washed twice with JC-1 staining buffer. Then JC-1 staining buffer (1 ml) was added to the cells, and was detected by flow cytometry.

### RNA Isolation and Quantitative Real-Time Polymerase Chain Reaction (qPCR)

Total RNA was collected using Trizol Reagent (Invitrogen, Carlsbad, CA, United States) according to the protocol. One microgram of total RNA was used to synthesize cDNA by reverse transcription using Revert Aid First Strand cDNA Synthesis Kit (Thermo Fisher Scientific, Waltham, MA, United States). Q-PCR test was performed by SYBR Green PCR Master Mix (Roche, Basle, Switzerland) using real-time PCR Detection System (ABI 7500, Life technology, United States). Target gene expression was normalized to the housekeeper gene GAPDH expression. The following primers were used: CYP2D6-F (5′-CCAGAAGGCTTTGCAGGCTTCA-3′) and CYP2D6-R (5′-ACTGAGCCCTGGGAGGTAGGTA-3′); CYP3A4-F (5′AAGAAACTGAGTCCCACAAAGC3′) and CYP3A4-R (5′ACACTGCTCGTGGTTTCACA3′); CYP3A5-F (5′TGTCCAGCAGAAACTGCAAA3′) and CYP3A5-R (5′TTGAAGAAGTCCTTGCGTGTC3′). To analyze the relative level of gene expression, we used the 2ˆ (-ΔΔCt) method.

### LC3 Immunocytochemistry

SH-SY5Y cells were cultured in 24-well plate at a density of 5 × 10^4^ cells/well. The 14 mm round coverslips coated with poly-L-lysine were added to the 24-well plate prior to seed cells. After treatment, coverslips were added with 4% paraformaldehyde for 20 min at room temperature. The paraformaldehyde was removed and washed by 0.01 M phosphate buffer (pH 7.4), then the coverslips were blocked in 10% normal goat serum with 0.3% Triton X-100 in PBS for 1 h at room temperature. Then, the coverslips were incubated with primary anti-LC3 antibody overnight at 4°C. Remove the primary antibody and wash the cells 3 × 3 min with PBS. The coverslips were incubated with Alexa Fluor^®^ 488 Conjugate secondary antibody for 1 h at room temperature. And the images were recorded and analyzed under a laser scanning confocal microscope (LMS710; Zeiss, Jena, Germany).

### TH Immunofluorescence

After rats were perfused with 120 ml of 4% paraformaldehyde in 0.01 M phosphate buffer (pH 7.4), the brains were harvested, postfixed in 4% paraformaldehyde for 6 ∼ 8 h, then transferred to 30% sucrose solution until the tissue to the bottom. The brain tissues were sectioned at a thickness of 20 μm. Briefly, the sections were permeabilized in PBST (containing 0.3% Triton X-100) for 10 min and blocked with 5% BSA in PBST for 1 h at room temperature. Then, the sections were incubated with primary anti-Tyrosine Hydroxylase antibody overnight at 4°C. The sections were then incubated with anti-rabbit secondary antibody for 1 h at room temperature. The images were captured by fluorescence microscopy (AXIO SCOPE A1, ZEISS).

### Western Blot Analysis

Protein samples were collected using RIPA lysates containing protease inhibitors and phosphatase inhibitors (Beyotime Biotechnology). To measure the protein concentration, the bicinchoninic acid assay (BCA) method with a total protein assay kit (Thermo Fisher) was used. The Biofuraw^TM^ Precast Gel (Tanon) was used to perform western blot, and the amount of each hole was 40 μg, and then electrophoretically transferred onto polyvinylidene difluoride membrane (PVDF) (Millipore), 100 V voltages for 90 min. The membranes were blocked with 5% non-fat milk in Tris-buffered saline containing 0.1% Tween-20 for 1 h at 37°C and then incubated with primary antibodies overnight at 4°C. The membranes were washed three times with Tris-buffered saline Tween-20 (TBST), and then incubated with horseradish peroxidase-conjugated secondary antibodies (Vazyme) for 1 h at 37°C and then a quantitative western blot images were recorded with fully automated chemiluminescence image analysis system (Tanon). Finally, densitometric analysis was performed with Image J software.

### Reagents

Morphine hydrochloride was supplied by Northeast Pharmaceutical Group Shenyang first pharmaceutical Co., Ltd. Naloxone hydrochloride was obtained from China National Medicines Guorui Pharmaceutical CO., Ltd. Rapamycin was obtained from Selleckchem. 6-OHDA, 4-PBA, 3-Methyladenine, apomorphine and DCFH-DA were purchased from Sigma. MTT assay kit and JC-1 were purchased from Beyotime Biotechnology. Annexin V/PI kit was purchased from BD Biosciences. The following antibodies were used: ATF6 (Abcam, ab37149), p-IRE1α (Novus Biologicals, NB100-2323), p-PERK (Biorbut, or b6693), AKT (Cell Signaling Technology, 9272), p-AKT(Cell Signaling Technology, 13038), IRE1α (Cell Signaling Technology, 3294), PERK (Cell Signaling Technology, 3192), Tyrosine Hydroxylase (Millipore, 2665965), CHOP (Cell Signaling Technology, 2895), GRP78 (Abcam, ab21685), DJ-1 (Cell Signaling Technology, 5933), β-actin (Immuno Way, YT0099), GAPDH (Immuno Way, YT5052), LC3 (Abcam, ab51520), p62 (Cell Signaling Technology, 8025), Parkin (Cell Signaling Technology, 4211), PINK1 (Cell Signaling Technology, 6946), Goat Anti-Mouse IgG (Vazyme, Ab201-03), Goat Anti-Rabbit IgG (Vazyme, Ab203-03), Anti-mouse IgG (Alexa Fluor^®^ 555 Conjugate) (Cell Signaling Technology, 4413), Anti-mouse IgG (Alexa Fluor^®^ 488 Conjugate) (Cell Signaling Technology, 4408).

### Statistical Analysis

Data were analyzed using Graph Prism 6 (Graph Pad, La Jolla, CA, United States). All values are presented as the mean ± SEM. The unpaired Student’s *t*-test was used for two-group comparisons. One-way ANOVA followed by the post-hoc Dunnett test was used for multiple comparisons. Differences with *P* < 0.05 were considered to be statistically significant.

## Results

### Morphine Protected SH-SY5Y Cells Against 6-OHDA-Induced Apoptosis

**FIGURE 1:**
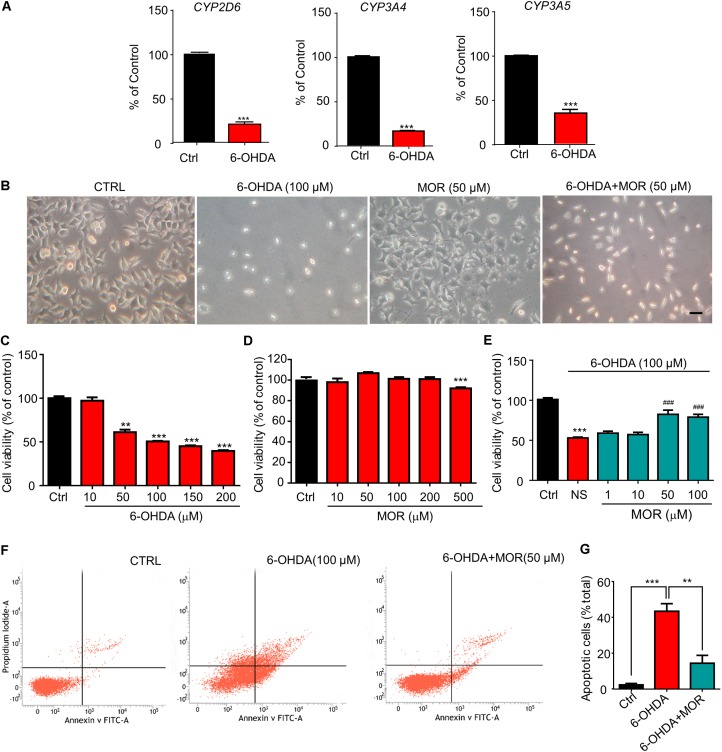
Morphine protected SH-SY5Y human neuroblastoma cells against 6-OHDA–induced cell apoptosis. **(A)** RT-qPCR analysis of the mRNA expression of *CYP2D6*, *CYP3A4* and *CYP3A5* following 6-OHDA treatment. ^∗∗∗^*P* < 0.001 vs. control. **(B)** The morphology of SH-SY5Y cells under phase contrast microscope. Scale bars: 50 μm. **(C)** Analysis of cell viability using the MTT assay in SH-SY5Y cells treated with 6-OHDA. *n* = 6–8 wells/group. **(D)** The effect of morphine on SH-SY5Y cells for 24 h by MTT assay. *n* = 7–8 wells/group. **(E)** The effect of pretreatment of low-dose morphine (1–100 μM) against the cytotoxicity induced by 6-OHDA (100 μM) in SH-SY5Y cells by MTT assay. ^∗∗^*P* < 0.01, ^∗∗∗^*P* < 0.001 vs. control group, ^###^*P* < 0.001 vs. 6-OHDA-treated group, *n* = 7–8 wells/group. **(F,G)** Flow cytometry was used to detect 6-OHDA-induced apoptosis with or without morphine. ^∗∗^*P* < 0.01, ^∗∗∗^*P* < 0.001 vs. 6-OHDA-treated group. All data are expressed as mean ± SEM.

Endogenous morphine synthesis was widely demonstrated in multiple tissues and cell types, including brain and SH-SY5Y cells (Muller et al., 2008; Laux-Biehlmann et al., 2013; Bodnar, 2018). Firstly, we explored the possible effects of 6-OHDA on the key enzymes involved in endogenous morphine biosynthesis, such as *CYP2D6*, *CYP3A4* and *CYP3A5* in SH-SY5Y cells (Lalovic et al., 2004). SH-SY5Y cells were incubated with 6-OHDA (100 μM) for 24 h, then the expression of mRNAs of *CYP2D6*, *CYP3A4* and *CYP3A5* were quantified by qPCR. Compared to the control cells, the expression of mRNAs of *CYP2D6*, *CYP3A4* and *CYP3A5* were significantly decreased by 80% (**Figure 1A**, *P* < 0.0001), 18% (**Figure 1A**, *P* < 0.0001) and 65% (**Figure 1A**, *P* = 0.0002) in 6-OHDA-treated cells. SH-SY5Y cells were seeded in 96-well plate and treated with different concentrations of 6-OHDA for 24 h. The viability of SH-SY5Y cells was analyzed using the MTT assay. As shown in **Figures 1B,C**, 6-OHDA treatment significantly reduced the viability of SH-SY5Y cells in a dose-dependent manner. 6-OHDA (100 μM) reduced the cell viability to about 50% (**Figure 1C**, *P* < 0.0001). Thus, 6-OHDA (100 μM) was chosen for the following experiments. As shown in **Figure 1D**, Morphine (10, 50, 100, and 200 μM) had no significant effect on viability of SH-SY5Y cells. However, high-dose morphine (500 μM) significantly reduced the viability of SH-SY5Y cells (**Figure 1D**). Then, we found morphine (50 μM) significantly attenuated cell injury induced by 6-OHDA (50% in 6-OHDA-treated group vs. 82% in morphine + 6-OHDA group compared with control group, **Figure 1E**, *P* < 0.0001). Thus, morphine (50 μM) was selected for the following experiments. Further, we quantified cell apoptosis by flow cytometry with Annexin V and PI double staining. 6-OHDA treatment significantly increased the percentage of apoptotic cells compared to the control group (43% in 6-OHDA-treated group vs. 2% in control group, **Figures 1F,G**, *P* = 0.0007). When cells were pretreated with morphine, the percentage of apoptotic cells induced by 6-OHDA was significantly reduced from 43 to 14% (**Figures 1F,G**, *P* = 0.0091). These results indicated morphine attenuated 6-OHDA-induced cell apoptosis, suggesting possible neuroprotective role of endogenous morphine in the cellular model of PD.

### Morphine Attenuated ER Stress Induced by 6-OHDA via Activation of the UPR

**FIGURE 2:**
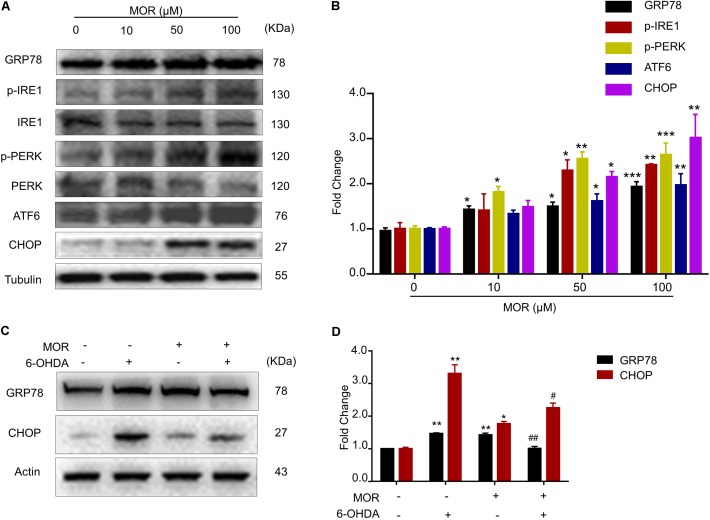
Morphine suppressed 6-OHDA-induced ER stress through activation of UPR. **(A,B)** Morphine induced UPR in SH-SY5Y cells. Protein levels of GRP78, p-IRE1α, IRE1α, p-PERK, PERK, ATF6, CHOP and Tubulin in SH-SY5Y cells were analyzed **(A)** and quantified **(B)** by western blot. ^∗^*P* < 0.05, ^∗∗^*P* < 0.01, ^∗∗∗^*P* < 0.001 vs. control. **(C,D)** Protein levels of GRP78, CHOP and Actin in SH-SY5Y cells were analyzed **(C)** and quantified **(D)** by western blot in the indicated groups. ^∗^*P* < 0.05, ^∗∗^*P* < 0.01 vs. control and ^#^*P* < 0.05, ^##^*P* < 0.01 vs. 6-OHDA-treated group. All data are expressed as mean ± SEM.

We subsequently examined the role of ER stress inhibition in morphine-induced neuroprotection *in vitro*. Firstly, we determined the effects of morphine on the three branches of UPR, including PERK, IRE1 and ATF6, and the downstream signaling including GRP78 and CHOP in SH-SY5Y cells. We found morphine (50 and 100 μM) significantly up-regulated the expression of all three UPR branches, including PERK, IRE1, ATF6, GRP78, and CHOP (**Figures 2A,B**), suggesting morphine activated the UPR activity. Then, we explored the effects of morphine on 6-OHDA-induced ER stress in SH-SY5Y cells. The results showed that 6-OHDA induced ER stress, which is reflected by significantly up-regulated GRP78 and CHOP expression in SH-SY5Y cells compared with control group (**Figures 2C,D**, *P* < 0.05). However, pretreatment of morphine significantly suppressed the up-regulation of GRP78 and CHOP induced by 6-OHDA (**Figures 2C,D**, *P* < 0.05). As described below, our results indicated that low-dose morphine may induce mild ER stress to triggered UPR activity for attenuating chronic ER stress, leading to neuroprotection against cell injury induced by 6-OHDA.

### Morphine Protected SH-SY5Y Cells Against 6-OHDA-Induced Apoptosis via Improvement of Autophagy

**FIGURE 3:**
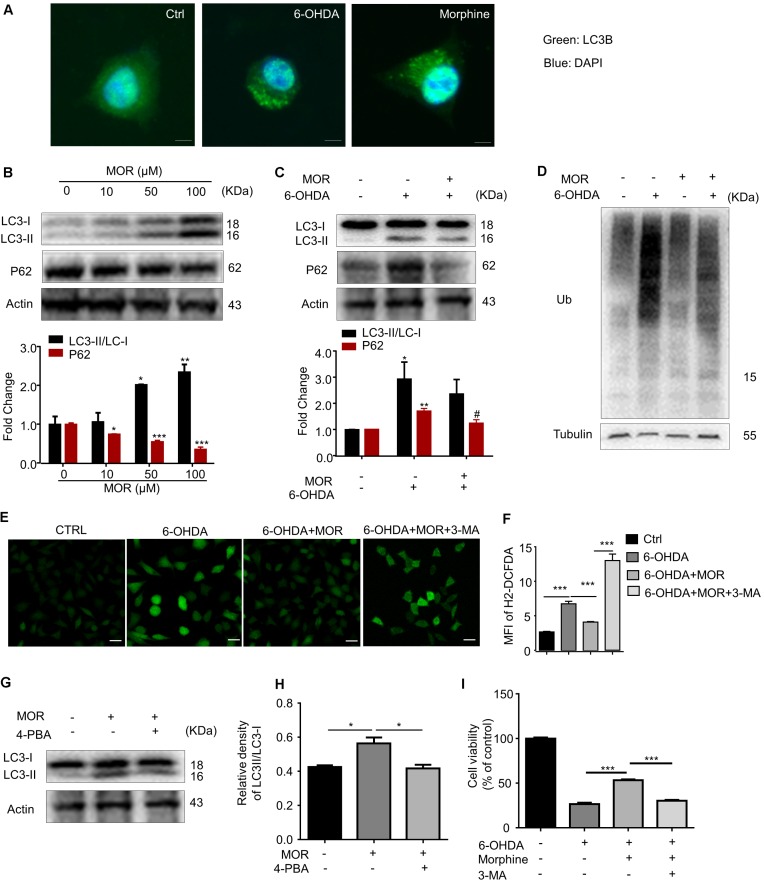
The role of autophagy in morphine-induced neuroprotection in SH-SY5Y cells. **(A)** Double immunofluorescent staining revealed accumulation of LC3 punctuated foci in cells exposed to 6-OHDA or morphine. Scale bars: 10 μm. **(B)** Protein levels of LC3, p62 and Actin in SH-SY5Y cells treated with morphine alone for 24 h were analyzed (upper panel) and quantified (lower panel) by western blot. ^∗^*P* < 0.05, ^∗∗^*P* < 0.01, ^∗∗∗^*P* < 0.001 vs. control. **(C)** The protein of LC3, p62 and Actin were analyzed (upper panel) and quantified (lower panel) by western blot. ^∗^*P* < 0.05, ^∗∗^*P* < 0.01 vs. control, ^#^*P* < 0.05 vs. 6-OHDA-treated group. **(D)** Western blotting showing the expression of ubiquitin (Ub) and Tubulin in SH-SY5Y cells pretreated with morphine for 24 h, followed by 6-OHDA treatment for 24 h. **(E,F)** The immunofluorescence **(E)** and quantified **(F)** the level of intracellular ROS by using DCFH-DA probe. ^∗∗∗^*P* < 0.001. Scale bars: 50 μm. **(G,H)** Protein levels of LC3 and Actin in SH-SY5Y cells pretreated with 4-PBA (2 mM) for 1 h, followed by morphine (50 μM) treatment for 24 h, then co-incubation with 6-OHDA (100 μM) for 24 h were analyzed **(G)** and quantified **(H)** by western blot. ^∗^*P* < 0.05, ^∗∗^*P* < 0.01, ^∗∗∗^*P* < 0.001 vs. control. **(I)** The effects of pretreatment of morphine (50 μM) with or without 3-MA (2 mM) on the cytotoxicity of 6-OHDA (100 μM) in SH-SY5Y cells were analyzed by MTT assay. 3-MA was added 1 h before morphine. ^∗∗∗^*P* < 0.001 vs. 6-OHDA+morphine-treated group. All data are expressed as mean ± SEM.

There are extensive connections between ER stress and autophagy (Rashid et al., 2015). We subsequently explored the role of activation of autophagy on morphine-induced neuroprotection *in vitro*. We observed that both 6-OHDA and morphine significantly increased the LC3 punctation in SH-SY5Y cells (**Figure 3A**), suggesting autophagy was induced by either 6-OHDA or morphine. Meanwhile, we found endogenous LC3 transformation into PE-conjugated LC3-II was dramatically increased after morphine treatment at 50 and 100 μM concentrations (**Figure 3B**, *P* < 0.05). In turn, morphine significantly decreased the expression of p62, a marker of autophagic degradation (**Figure 3B**, *P* < 0.05). These results indicated morphine activated autophagy in SH-SY5Y cells, which was consistent with previous study (Dever et al., 2015). Although 6-OHDA increased LC3-II level in SH-SY5Y cells (**Figure 3C**, *P* < 0.05), it failed to decrease the level of p62, suggesting that autophagic influx was impaired by 6-OHDA treatment. Next, we investigated effect of morphine on the impairment of autophagic influx induced by 6-OHDA. Interestingly, morphine significantly decreased the expression of p62 in 6-OHDA-treated SH-SY5Y cells (**Figure 3C**, *P* < 0.05), suggesting morphine improved 6-OHDA-induced the impairment of autophagic influx. Accordingly, we also found morphine reduced the accumulation of ubiquitinated proteins, which was remarkably increased after 6-OHDA treatment (**Figure 3D**). We further found that morphine significantly suppressed the increased intracellular calcium level in 6-OHDA-treated SH-SY5Y cells (**Figures 3E,F**, *P* = 0.0004), which was partially reversed by an autophagy inhibitor 3-MA (**Figures 3E,F**, *P* < 0.0001). In addition, morphine-induced up-regulation of LC3-II was abolished by a chemical chaperone and UPR inhibitor, 4-phenylbutyrate (4-PBA) (**Figures 3G,H**), indicating morphine-induced the activation of autophagy may be attributed to the activation of UPR. Finally, we found 3-MA abolished the neuroprotective effect of morphine in 6-OHDA-treated SH-SY5Y cells (**Figure 3I**, *P* < 0.001). Together, these data indicated that morphine improved the impaired of autophagy induced by 6-OHDA and played an important role in morphine’s neuroprotective effects in SH-SY5Y cells.

### μ-opioid Receptor and AKT/mTOR Pathways Were Involved in Morphine-Induced Neuroprotection

**FIGURE 4:**
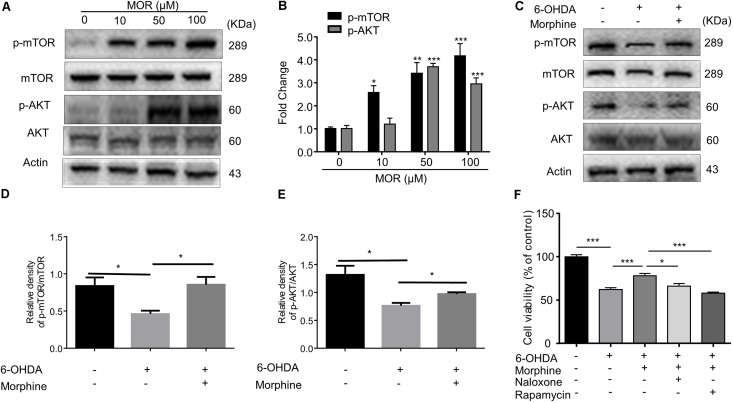
The role of AKT/mTOR pathway in morphine-induced neuroprotection in SH-SY5Y cells. **(A,B)** Protein levels of p-mTOR, mTOR, p-AKT, AKT and Actin in SH-SY5Y cells with morphine (10, 50, and 100 μM) alone for 24 h were analyzed **(A)** and quantified **(B)** in the indicated groups. ^∗^*P* < 0.05, ^∗∗^*P* < 0.01, ^∗∗∗^*P* < 0.001 vs. control. **(C–E)** Protein levels of p-mTOR, mTOR, p-AKT, AKT and actin in SH-SY5Y cells pretreated with rapamycin (1 μM) for 3 h, followed by morphine (50 μM) treatment for 24 h, then co-incubation with 6-OHDA (100 μM) for 24 h were analyzed **(C)** and quantified **(D,E)** in the indicated groups. ^∗^*P* < 0.05, ^∗∗^*P* < 0.01 vs. control. **(F)** The effects of pretreatment of morphine (50 μM) with or without rapamycin (1 μM) and naloxone (10 μM) on the cytotoxicity of 6-OHDA (100 μM) in SH-SY5Y cells were analyzed by MTT assay. Rapamycin (1 μM) or naloxone (10 μM) were added 3 h before morphine. ^∗^*P* < 0.05, ^∗∗∗^*P* < 0.001. All data are expressed as mean ± SEM.

Although previous report found that morphine induced autophagy in ATG5 and Beclin1-dependent manner (Zhao et al., 2010), it is still unclear whether mTOR pathway was involved in morphine-induced neuroprotection or not. As shown in **Figures 4A,B**, morphine (50 and 100 μM) significantly induced phosphorylation of AKT and mTOR in SH-SY5Y cells. Meanwhile, pretreatment of morphine significantly restored the decreased expression of phosphorylated AKT and mTOR induced by 6-OHDA (**Figures 4C–E**, *P* < 0.01). Given mTOR inhibition is considered to trigger autophagy, our results indicated morphine-induced autophagy was independent on mTOR inhibition. Treatment with μ-opioid receptor antagonist naloxone and mTOR inhibitor rapamycin abolished the neuroprotective effects of morphine (**Figure 4F**), indicating activation of μ-opioid receptor and mTOR signaling contributes to the neuroprotective effects of morphine. Thus, these data indicated μ-opioid receptor and AKT/mTOR signaling are involved in morphine-induced neuroprotection.

### Morphine Improved Mitochondria Dysfunction and Suppressed the Accumulation of Intracellular ROS

**FIGURE 5:**
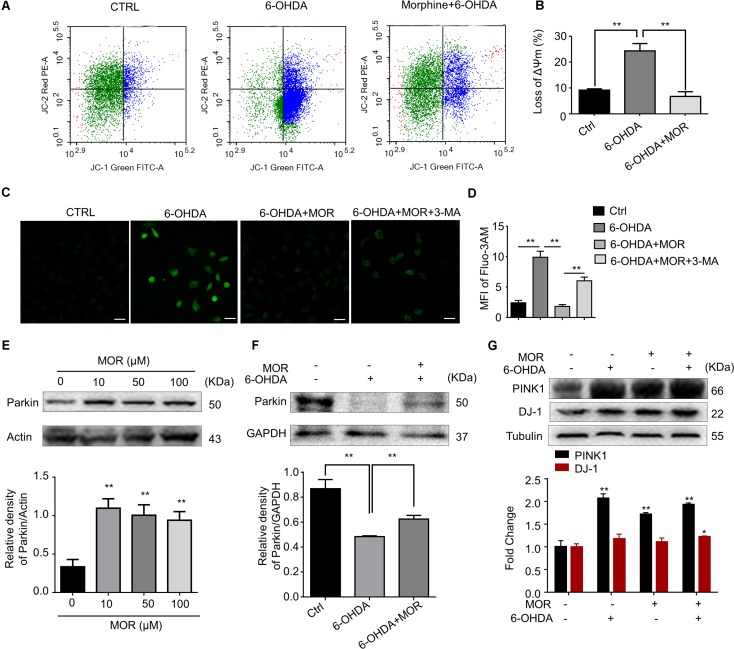
Morphine improved mitochondria function in SH-SY5Y cells. **(A,B)** JC-1 staining and flow cytometry were used to analyze **(A)** and quantify **(B)** changes in mitochondrial membrane potential. ^∗∗^*P* < 0.01 vs. 6-OHDA-treated group. **(C,D)** Immunofluorescence of Fluo-3/AM was used to analyze **(C)** and quantify **(D)** the level of intracellular calcium concentration. ^∗∗^*P* < 0.01. Scale bars: 50 μm. **(E)** Protein levels of Parkin and Actin in SH-SY5Y cells with morphine alone for 24 h were analyzed (upper panel) and quantified (lower panel) by Western blot analysis. ^∗∗^*P* < 0.05 vs. control. **(F)** Protein levels of Parkin and Actin in SH-SY5Y cells were analyzed (upper panel) and quantified (lower panel) by Western blotting analysis in the indicated groups. ^∗∗^*P* < 0.01, ^∗∗∗^*P* < 0.001 vs. 6-OHDA-treated group. **(G)** The protein levels of PINK1, DJ-1 and Tubulin in SH-SY5Y cells were analyzed (upper panel) and quantified (lower panel) by Western blot analysis. ^∗^*P* < 0.05, ^∗∗^*P* < 0.01 vs. control. All data are expressed as mean ± SEM.

**FIGURE 6:**
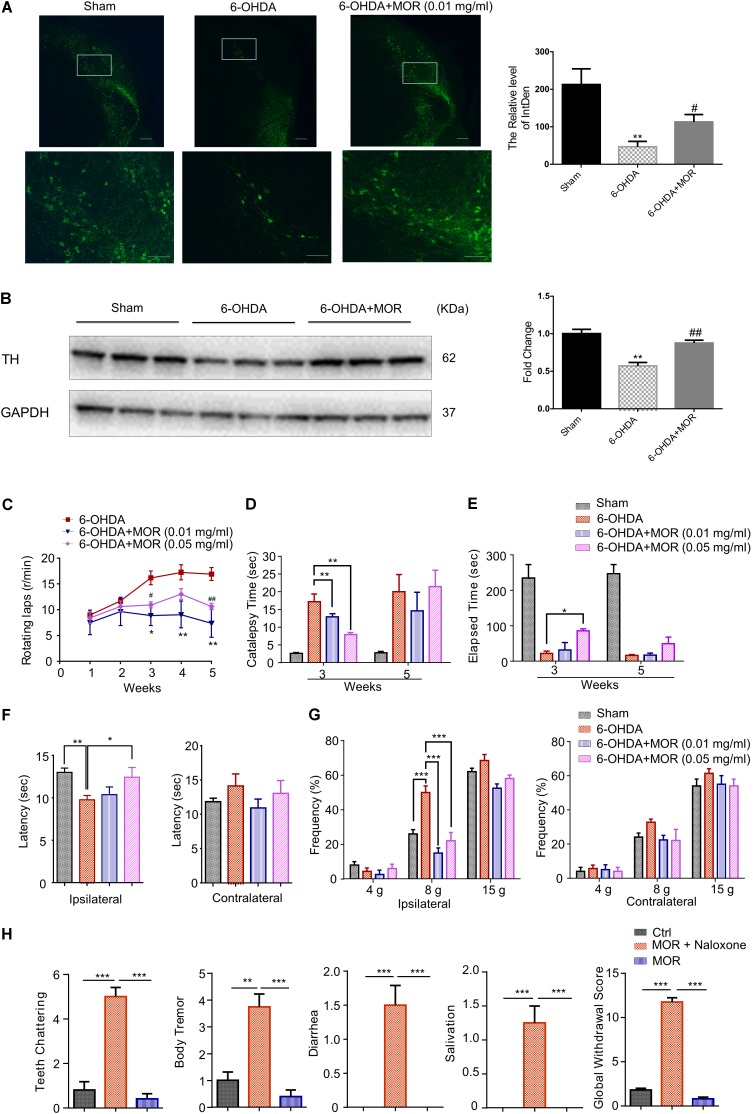
Morphine reduced the loss of dopaminergic neurons, improved motor dysfunction and inhibited pain hypersensitivity in 6-OHDA-lesioned rats. **(A)** Left panel is immunofluorescence of TH (green) in the SNc. Right panel is quantification of TH^+^ nerve fibers. Scale bars: 500 μm (upper panel) and 100 μm (lower panel). **(B)** TH expression in SNc was analyzed (left panel) and semi-quantitatively determined (right panel) by western blotting. ^∗^*P* < 0.05, ^∗∗∗^*P* < 0.001 vs. 6-OHDA treated group, *n* = 4–6. **(C)** Oral low-dose morphine reduced apomorphine-induced rotation in 6-OHDA-treated rats. ^∗^*P* < 0.05, ^∗∗^*P* < 0.01 vs. 6-OHDA-treated group, ^#^*P* < 0.05, ^##^*P* < 0.01 vs. 6-OHDA-treated group. *n* = 4–6. **(D)** The stiffness of PD rats was significantly improved by oral morphine at the 3rd week after operation by catalepsy test. **(E)** The impaired motor function of PD rats was significantly improved after oral morphine on the 3rd week after operation by rotarod test. **(F)** Paw thermal withdrawal latency of rats was tested by Hargreaves testing. **(G)** Paw mechanical withdrawal threshold of rats was determined by von Frey filaments (4, 8, and 15 g). ^∗^*P* < 0.05, ^∗∗^*P* < 0.01, ^∗∗∗^*P* < 0.001 vs. 6-OHDA-treated group. **(H)** Naloxone-induced withdrawal symptoms in oral morphine-treated 6-OHDA-lesioned rats. ^∗∗^*P* < 0.01, ^∗∗∗^*P* < 0.001 vs. morphine addiction model group, *n* = 4–6 rats per group. All data are expressed as mean ± SEM.

Mounting evidence supported mitochondria dysfunction contributes to the pathogenesis of PD (Pickrell and Youle, 2015). We measured mitochondrial membrane potential by using JC-1 staining in SH-SY5Y cells. SH-SY5Y cells were pretreated with morphine (50 μM) for 24 h, and then treated with 6-OHDA (100 μM) for another 24 h. As shown in **Figures 5A,B**, 6-OHDA treatment induced significant loss of mitochondrial membrane potentials in SH-SY5Y cells compared with control cells (*P* = 0.0057), while morphine significantly prevented 6-OHDA-induced loss of mitochondrial membrane potentials (*P* = 0.0058). Mitochondria are considered as the main source for generation of intracellular reactive oxygen species (ROS) (Diebold and Chandel, 2016). Furthermore, we found the pretreatment of morphine significantly suppressed the increased accumulation of intracellular ROS in 6-OHDA-treated SH-SY5Y cells (**Figures 5C,D**, *P* = 0.0004), which was abolished by 3-MA, an autophagy inhibitor (**Figures 5C,D**, *P* < 0.0001). In addition, recent study indicated mitophagy mediated by PINK1-Parkin pathway played important roles in the maintenance of mitochondrial activity (Rodolfo et al., 2017). Incubation of morphine (10, 50, and 100 μM) alone significantly increased the expression of Parkin in SH-SY5Y cells (**Figure 5E**, *P* < 0.05). In contrast, 6-OHDA treatment significantly decreased the expression of Parkin (**Figure 5F**, *P* < 0.05). Interestingly, morphine significantly restored the expression of Parkin in 6-OHDA-treated cells (**Figure 5E**, *P* < 0.05). 6-OHDA treatment significantly increased the expression of PINK1 (**Figure 5G**, *P* < 0.05), while morphine was not able to further increase the expression of PINK1 in SH-SY5Y cells. Additionally, morphine also significantly increased the expression of DJ-1 in 6-OHDA-treated cells (**Figure 5G**, *P* < 0.05). Together, these results indicated morphine may improve PINK1/Parkin-mediated mitophagy, which was impaired by 6-OHDA.

### Oral Administration of Low-Dose Morphine Improved the Neurochemical and Behavioral Deficits Induced by 6-OHDA in Rats

According to previous report (Sun et al., 2014), the rat model of PD was established by micro-injection of 6-OHDA (2 μg/μl in 3 μl) into the right medial forebrain bundle. We examined the possible neuroprotective effects of oral low-dose morphine in this 6-OHDA-induced PD model. Oral application of low-dose morphine (0.01 mg/ml) restored 6-OHDA-induced decreased intensity of TH immunofluorescence staining (**Figure 6A**). As shown in **Figure 6B**, western blotting analysis showed that the expression of TH was significantly reduced by 32% in SNc of 6-OHDA-lesioned rats compared with sham group (*P* < 0.0001). Interestingly, oral low-dose morphine significantly increased TH expression in SNc of 6-OHDA-lesioned rats (**Figure 6B**, *P* = 0.014). Apomorphine-induced rotation in 6-OHDA-lesioned rats was to determine the successful establishment of PD rat model and behaviorally examine the neuroprotective effects of morphine in PD rats. Apomorphine is a dopamine receptor agonist, while morphine is opioid receptor agonist. Thus, apomorphine is a distinct compound that does not mimic the receptor-mediated effects of morphine. The 6-OHDA-lesioned rats exhibited robust contralateral turning after apomorphine administration from 1 to 5 weeks after 6-OHDA injection in the right medial forebrain bundle (**Figure 6C**). Moreover, oral morphine (0.01 mg/ml) significantly reduced apomorphine-induced rotation in 6-OHDA-lesioned rats at the 3rd, 4th, and 5th week after 6-OHDA injection (**Figure 6C**, *P* < 0.05). We found oral morphine (0.05 mg/ml) significantly reduced apomorphine-induced rotation in 6-OHDA-lesioned rats at 3rd and 5th week (**Figure 6C**, *P* < 0.05). Catalepsy behavior was assessed 3 and 5 weeks after 6-OHDA injection. The duration of catalepsy was remarkably increased in 6-OHDA-lesioned rats compared with sham rats at 3rd week (17 s in 6-OHDA-lesioned group vs. 2.6 s in sham group; **Figure 6D**, *P* < 0.0001). Interestingly, oral morphine (0.01 mg/ml and 0.05 mg/ml) attenuated the duration of catalepsy behavior induced by 6-OHDA at the 3rd week after 6-OHDA injection (17 s in 6-OHDA-lesioned group vs. 12 s in 0.01 mg/ml morphine group, *P* = 0.0085 and 7 s in 0.05 mg/ml morphine group, *P* = 0.0014; **Figure 6D**), but not at 5th week after 6-OHDA injection. In addition, we assessed the motor coordination by Rota-rod test. We found morphine (0.05 mg/ml) improved motor function in the 6-OHDA-lesioned rats at 3rd week after 6-OHDA injection (**Figure 6E**, *P* = 0.0484), but not at the 5th week after 6-OHDA injection. Meanwhile, we demonstrated morphine attenuated thermal and mechanical pain hypersensitivity in the 6-OHDA-lesioned rats at 3rd week after 6-OHDA injection (**Figures 6F,G**). Finally, we found that naloxone induced behavioral signs of withdrawal when morphine was intraperitoneally given twice every day for 5 days, including teeth-chattering, body tremor, and diarrhea in rats (**Figure 6H**). In sharp contrast, we found naloxone did not induce apparent behavioral signs of withdrawal in rats that was oral administrated of 0.01 mg/ml morphine (**Figure 6H**), suggesting oral administrated of low-dose morphine may not result in physical dependence.

### Morphine Prolonged the Lifespan and Improved Motor Function in Transgenic PD Drosophila Models

**FIGURE 7:**
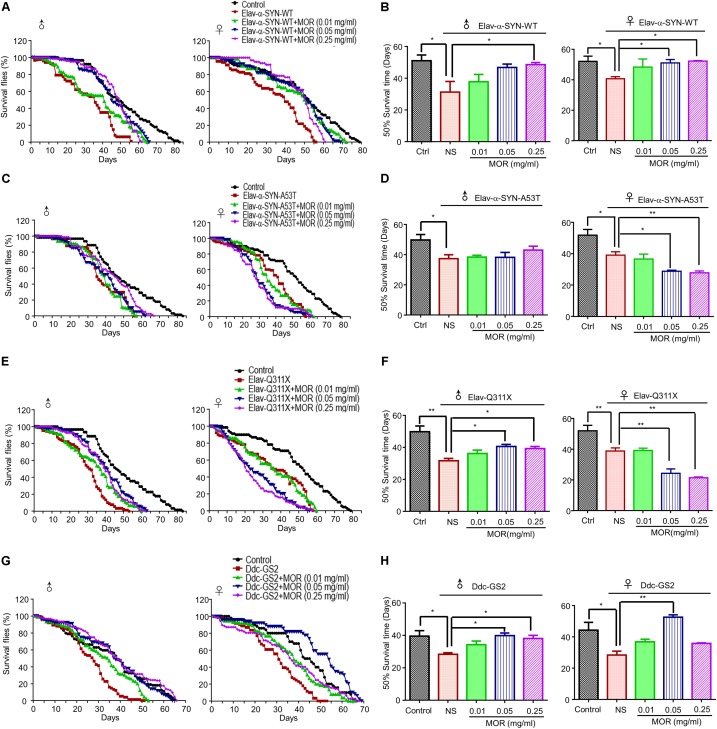
Effects of low-dose morphine on the lifespan of transgenic PD *Drosophila* models. The lifespan of adult male and female *Drosophila* of different genotypes were recorded at 25°C. A minimum of one hundred flies for each group were analyzed, and the number of surviving flies were counted daily, and survivorship curves were generated. **(A,B)** Morphine prolonged the lifespan of *Elav-α-synuclein-WT* transgenic PD flies. ^∗^*P* < 0.05 vs. *Elav-α-synuclein-WT* group, *n* = 80–120 flies/group. **(C,D)** Morphine had no effect on the lifespan of male flies in *Elav-α-synuclein* genotype, but can shorten the lifespan of female flies. ^∗^*P* < 0.05, ^∗∗^*P* < 0.01 vs. *Elav-α-synuclein-A53T* group, *n* = 80–120 flies/group. **(E,F)** Morphine prolonged the lifespan of *Elav-Q311X* transgenic PD male flies, but shortened the lifespan of female flies. ^∗^*P* < 0.05 vs. *Elav-Q311X* group, *n* = 80–120 flies/group. **(G,H)** Morphine prolonged the lifespan of *Ddc-GS2* transgenic PD male flies, while only morphine (0.05 mg/ml) prolonged the lifespan of female flies. ^∗^*P* < 0.05, ^∗∗^*P* < 0.01 vs. *Ddc-GS2* group, *n* = 80–120 flies/group. All data are expressed as mean ± SEM.

As previously reported (Wang B. et al., 2015), PD-like neurodegenerative phenotypes were demonstrated in *Elav-α-synuclein-WT*, *Elav-α-synuclein-A53T*, *Elav-Q311X* (*Parkin* mutation) and *Ddc-GS2* (*LRRK2* mutation) transgenic fruit fly (*Drosophila* melanogaster). As shown in **Figure 7**, we observed shorter lifespan in all 4 transgenic lines of male and female flies compared with wild-type (WT) controls. We feed transgenic flies with morphine (0.01 mg/ml, 0.05 mg/ml, and 0.25 mg/ml). Morphine (0.25 mg/ml) treatment significantly prolonged the 50% survival time by about 18, 9 and 8 days in *Elav-α-synuclein-WT* (**Figures 7A,B**, *P* = 0.033), *Elav-Q311X* (**Figures 7E,F**, *P* = 0.0052) and *Ddc-GS2* (**Figures 7G,H**, *P* = 0.0118) genotype male flies compared to control male flies, respectively. Morphine (0.05 mg/ml) treatment significantly prolonged the 50% survival time by 8 and 11 days in *Elav-Q311X* (**Figures 7E,F**, *P* = 0.0019) and *Ddc-GS2* (**Figures 7G,H**, *P* = 0.0028) genotype male flies compared to control male flies, respectively. However, we found that morphine could not prolong the 50% survival time in male *Elav-α-synuclein-A53T* flies (**Figures 7C,D**). Morphine (0.05 mg/ml) significantly prolonged the 50% survival time in *Elav-α-synuclein-WT* (**Figures 7A,B**, *P* < 0.05) and *Ddc-GS2* (**Figures 7G,H**, *P* < 0.01) genotype female flies compared to control female flies, respectively. Unexpected, morphine (0.05 mg/ml and 0.25 mg/ml) shortened the 50% survival time of female *Elav-α-synuclein-A53T* (**Figures 7C,D**) and *Elav-Q311X* flies (**Figures 7E,F**).

**FIGURE 8:**
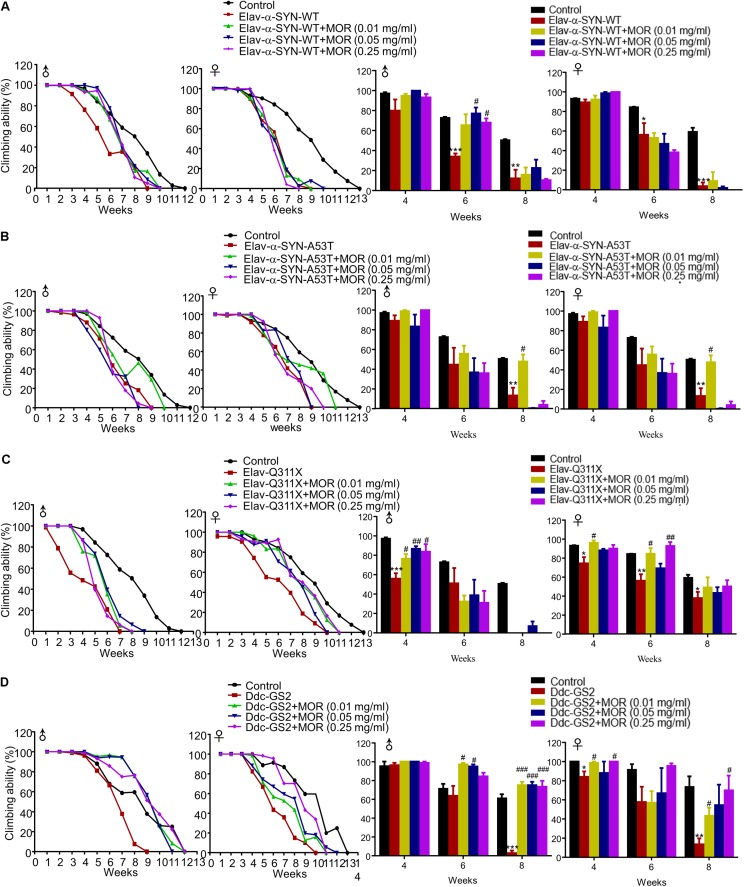
Effects of low-dose morphine on climbing behavior of transgenic PD *Drosophila* models. **(A–D)** Effects of low-dose morphine on the climbing abilities of *Elav-α-synuclein-WT* **(A)**, *Elav-α-synuclein-A53T* **(B)**, *Elav-Q311X* **(C)**, *Ddc-GS2* **(D)** transgenic *Drosophila*. ^∗^*P* < 0.05, ^∗∗^*P* < 0.01, ^∗∗∗^*P* < 0.001 vs. control, ^#^*P* < 0.05, ^##^*P* < 0.01, ^###^*P* < 0.001 vs. transgenic PD flies, *n* = 80–120 flies/group. All data are expressed as mean ± SEM.

Additionally, we found morphine improved climbing behavior in *Elav-α-synuclein-WT*, *Elav-Q311X* and *Ddc-GS2* genotype male flies, but not for *Elav-α-synuclein-A53T* genotype male flies (**Figure 8**). Morphine significantly improved the climbing activity in *Elav-Q311X* and *Ddc-GS2* genotype female flies, but not in *Elav-α-synuclein-WT* and *Elav-α-synuclein-A53T* genotype female flies (**Figure 8**). Thus, these data suggested that gender, genotype, and dosage dramatically affected the neuroprotective effects of morphine in transgenic *Drosophila* models of PD.

### Morphine Prolonged the Lifespan and Improved Motor Function in Rotenone-Induced PD Drosophila Model

**FIGURE 9:**
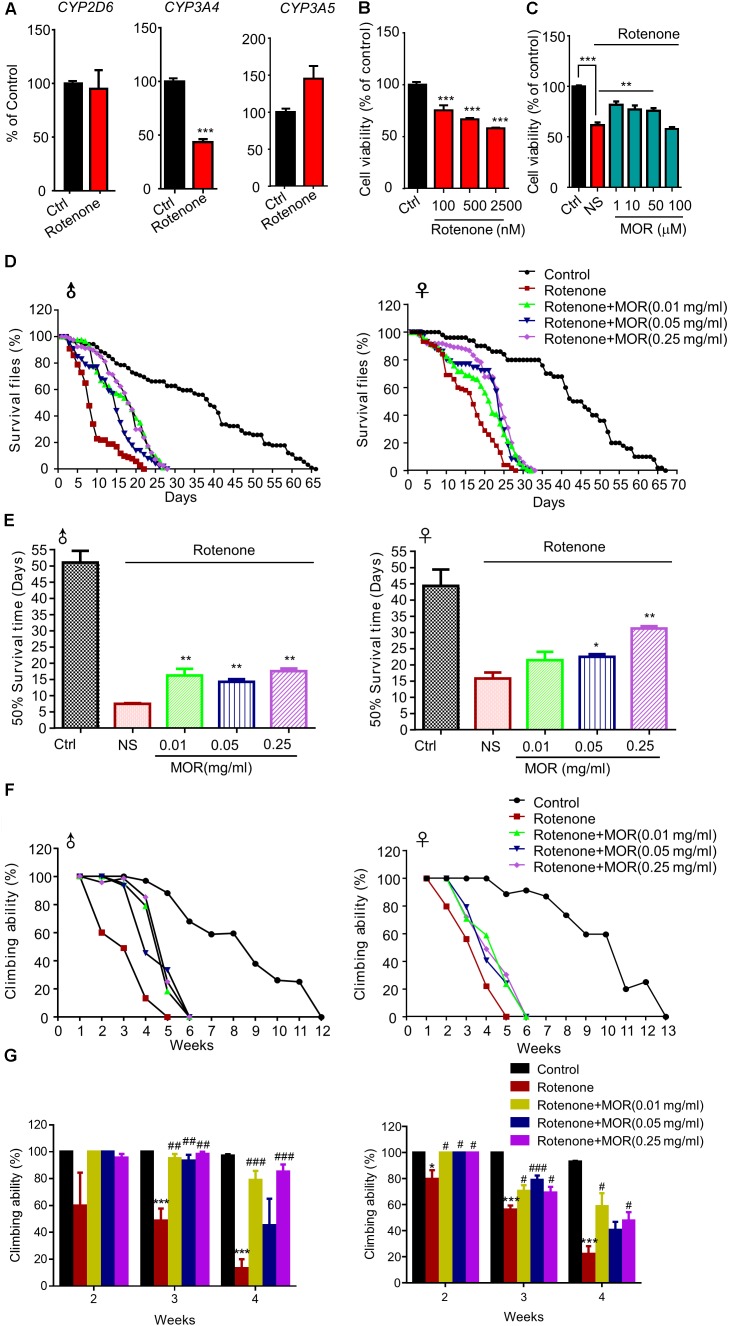
Effects of low-dose morphine on rotenone-induced cellular and animal models. **(A)** RT-qPCR analysis of the mRNA expression of *CYP2D6*, *CYP3A4* and *CYP3A5*. ^∗∗∗^*P* < 0.001 vs. control. **(B)** Analysis of cell viability using the MTT assay in SH-SY5Y cells treated with rotenone. *n* = 6–8 wells/group. ^∗∗∗^*P* < 0.001. **(C)** The effect of pretreatment of morphine (1–100 μM) on the cytotoxicity of rotenone (100 μM) in SH-SY5Y cells by MTT assay. *n* = 6–8 wells/group. ^∗∗^*P* < 0.01, ^∗∗∗^*P* < 0.001. **(D,E)** Morphine prolonged the lifespan of rotenone-induced *Drosophila* model. **(F,G)** Morphine improved the motor function of rotenone-induced *Drosophila* model. ^∗^*P* < 0.05, ^∗∗^*P* < 0.01, ^∗∗∗^*P* < 0.001 vs. control and ^#^*P* < 0.05, ^##^*P* < 0.01, ^###^*P* < 0.001 vs. rotenone-treated *Drosophila*, *n* = 80–120 flies/group. All data are expressed as mean ± SEM.

Finally, we explored the neuroprotective effects of morphine in rotenone-induced cellular and animal PD models. We found rotenone significantly decreased CYP3A4 mRNA, a key enzyme for endogenous morphine (**Figure 9A**, *P* < 0.05). Rotenone significantly decreased the cell viability in SH-SY5Y cells dose-dependently (**Figure 9B**, *P* < 0.05). Pretreatment of low-dose morphine (1, 10, and 50 μM) significantly attenuated cell injury induced by rotenone (100 μM) (**Figure 9C**, *P* < 0.05). We found rotenone (100 μM) significantly decreased the 50% survival time to 44 and 29 days in male and female flies, respectively (**Figures 9D,E**). Interestingly, feed with low-dose morphine (0.01 mg/ml) prolonged 50% survival time of male flies, while had no effect in female flies (**Figures 9D,E**). Morphine (0.05 mg/ml and 0.25 mg/ml) prolonged 50% survival time of male and female flies (**Figures 9D,E**). We also demonstrated that morphine significantly improved the climbing behavior, which was impaired by rotenone (**Figures 9F,G**). Thus, low dose morphine also produced neuroprotection in rotenone-induced PD models.

## Discussion

The present study aimed to explore the neuroprotective effects of low-dose morphine in the cellular and animal PD models and possible underlying mechanisms. Our results showed that pretreatment with morphine attenuated cellular injury induced by 6-OHDA or rotenone, which are well-known PD-related toxins. Furthermore, we revealed that attenuation of ER stress and oxidative stress, activation of autophagy and improvement of mitochondrial function contribute to the neuroprotective effects of morphine in the cellular PD model. Moreover, oral application of low-dose morphine improved neurochemical and behavioral deficits in 6-OHDA-lesioned rats without sign of physical dependence and prolonged the lifespan and improved motor function in rotenone-induced and transgenic *Drosophila* PD models. Together, our present study demonstrated that low-dose morphine may be clinical useful neuroprotective drug for PD treatment.

ER controls the quality of proteins and maintains protein homeostasis via modulating intracellular calcium levels and the folding of proteins synthesized in the cells (Wang and Kaufman, 2016). Accumulated unfolded/misfolded proteins in ER activate UPR such as ATF6, IRE1 and PERK to attenuate ER stress. The activation of UPR help to correct the protein folding function. However, once the accumulation of unfolded proteins surpasses the corrective function, cell death will occur (Walter and Ron, 2011). Many reports suggested ER stress contributed to the pathogenesis of PD (Remondelli and Renna, 2017). For instance, GRP78 and CHOP, two ER stress markers, were increased in MPP^+^-treated cells and MPTP-induced PD mouse model (Tsujii et al., 2015; Xiao et al., 2016). Accordingly, we also found the expression of GRP78 and CHOP were increased in SH-SY5Y cells treated with 6-OHDA. The pretreatment of morphine can decrease the expression of GRP78 and CHOP. Consistent with previous study (Cai et al., 2016), we found that 3 branches of UPR (including PERK, IRE1 and ATF6) were activated and the downstream effectors including GRP78 and CHOP were increased, when SH-SY5Y cells were treated with low-dose morphine. Thus, we concluded that low-dose morphine may induce mild ER stress to triggered UPR activity for attenuating chronic ER stress, leading to neuroprotection against cell injury induced by PD-related neurotoxins. We also proposed a hypothesis that low-dose morphine activates a particular signaling pathway to induce UPR, instead of an indirect effect reflecting cell viability. The precise mechanisms underlying morphine-induced UPR activity need further investigation.

Compelling evidence supports ER stress had crosstalk with autophagy (Nakka et al., 2016). Autophagy mediates the degradation of misfolded/aggregated proteins and dysfunctional organells in cells, and plays an important role in the maintenance of cellular homeostasis (Shen et al., 2015; García-Prat et al., 2016). Mounting evidence suggested that autophagy dysfunction resulted in the accumulation of misfolded or aggregated proteins and damaged organelles, which were commonly observed in neurodegenerative diseases, such as Alzheimer’s disease (AD) and PD (Yang et al., 2011; Rui et al., 2015; Zhang et al., 2015). In the present study, we found 6-OHDA increased the expression of LC3-II, a marker of autophagosome, but failed to decrease p62, a substrate of autophagy. These results indicated 6-OHDA induced impairment of autophagic flux in cells. Meanwhile, morphine treatment induced the up-regulation of LC3-II and facilitated the degradation of autophagic substrate, p62. Furthermore, morphine improved the impaired autophagic flux induced by 6-OHDA. Other studies confirmed that morphine triggered autophagy (Dever et al., 2015; Cai et al., 2016). It is well-known that mTOR is a typical suppressor of autophagy (Kim and Guan, 2015). We found that morphine activated AKT-mTOR signaling pathway, which was consistent with previous studies (Wang Y. et al., 2015; Jin et al., 2017). Thus, these data indicated the activation of autophagy triggered by morphine was independent of mTOR pathway and instead it may be due to up-regulation of Becin-1 and ATG-5/7 (Zhao et al., 2010). Together, we demonstrated activation of autophagy played a critical role in morphine-induced attenuation of ER stress and neuroprotection for PD.

Mitochondrial dysfunction is also considered as critical mechanism underlying the pathogenesis of PD (Ryan et al., 2015; Bose and Beal, 2016). In the current study, we showed that pretreatment of morphine significantly increased the loss of mitochondrial membrane potential by 6-OHDA. As mitochondria are the major sources of intracellular ROS production, our results showed 6-OHDA remarkably promoted intracellular ROS accumulation. Interestingly, morphine significantly reduced intracellular ROS production induced by 6-OHDA. Damaged mitochondria are mainly removed by autophagic degradation, termed mitophagy. PINK1-dependent activation of Parkin is recognized as a major pathway to induce mitophagy (Eiyama and Okamoto, 2015). Mitophagy is essential for the control of mitochondrial quality, as mitophagy impairment will result in the persistence of damaged mitochondria and intracellular ROS accumulation. The mutation of PINK1 or Parkin causes early onset PD (Weissbach et al., 2017). We found that expression of PINK1 was significantly up-regulated and Parkin was significantly decreased after 6-OHDA treatment. Interestingly, morphine restored the expression of Parkin which was inhibited by 6-OHDA. Thus, these data suggested morphine may promote mitophagy for removing damaged mitochondria and inhibiting accumulation of intracellular ROS.

Previous study demonstrated endogenous morphine presents in human SH-SY5Y neuroblastoma cell line (Muller et al., 2008), which is often used for PD study *in vitro*. Stefano et al. (2012) reported endogenous morphine biosynthesis was dysregulated in PD. The key enzymes for endogenous morphine biosynthesis include *CYP2D6*, *CYP3A4* and *CYP3A5*. In the present study, we found that 6-OHDA decreased the mRNA expression level of *CYP2D6*, *CYP3A4* and *CYP3A5* in SH-SY5Y cells. In addition, rotenone also decreased the mRNA expression of CYP3A4 in SH-SY5Y cells. Thus, these data indicated endogenous morphine biosynthesis was impaired in cellular PD model. To answer the question whether supply of low-dose morphine is beneficial for PD, we explored the neuroprotective effects of oral feeding of low doses of morphine in 6-OHDA-induced rat PD model and several transgenic and rotenone-induced *Drosophila* PD models. These results showed that oral administration of low-dose morphine prevented the loss of DA neurons in midbrain and improved the motor deficits in 6-OHDA-induced rat PD model. Treatment of pain in PD patients remains a challenging problem in clinic (Beiske et al., 2009). Interestingly, our results demonstrated morphine also inhibited mechanical and thermal pain hypersensitivity in PD rats, suggesting morphine may be beneficial for pain relief in PD patients. Additionally, low-dose morphine did not induce behavioral signs of physical dependence in rats. For transgenic *Drosophila* PD models, feeding of low-dose morphine produced mixed effects on the lifespan and motor function in gender, genotype, and dose-dependent manner. For male flies, morphine prolonged the lifespan of flies with *Elav-α-synuclein-WT*, *Elav-Q311X* and *Ddc-GS2* genotypes, but not *Elav-α-synuclein-A53T* genotype. For female flies, morphine prolonged the lifespan of flies with *Elav-α-synuclein-WT* and *Ddc-GS2* genotypes, but oppositely shortened the lifespan of flies with *Elav-Q311X* and *Elav-α-synuclein-A53T* genotypes. In female flies with *Ddc-GS2* genotype, the dose-response curve of morphine is “invert-U” shape. However, how sex and genotypes affect the neuroprotection of morphine is still unclear and warrants further investigation.

In summary, we demonstrated low-dose morphine produced neuroprotective effects in cellular and animal PD models, possible through activation of autophagy, attenuation of ER stress and oxidative stress, and improvement of mitochondrial functions. From the results of transgenic *Drosophila* PD models, we also found that neuroprotective effects of morphine were dependent on sex, genotype, and dosage. Thus, we suggested that low-dose morphine may be a promising neuroprotective drug for PD therapy.

## Author Contributions

All authors listed have made a substantial, direct and intellectual contribution to the work, and approved it for publication.

## Conflict of Interest Statement

The authors declare that the research was conducted in the absence of any commercial or financial relationships that could be construed as a potential conflict of interest.
